# The Role of Iron in Cancer Progression

**DOI:** 10.3389/fonc.2021.778492

**Published:** 2021-11-10

**Authors:** Qianqian Guo, Liwen Li, Shanshan Hou, Ziqiao Yuan, Chenhui Li, Wenzhou Zhang, Lufeng Zheng, Xiaoman Li

**Affiliations:** ^1^ Department of Pharmacy, Affiliated Cancer Hospital of Zhengzhou University, Henan Cancer Hospital, Zhengzhou, China; ^2^ Jiangsu Key Laboratory for Pharmacology and Safety Evaluation of Chinese Materia Medica, School of Pharmacy, Nanjing University of Chinese Medicine, Nanjing, China; ^3^ School of Life Science and Technology, Jiangsu Key Laboratory of Carcinogenesis and Intervention, School of Basic Medicine and Clinical Pharmacy, China Pharmaceutical University, Nanjing, China; ^4^ Department of Pharmacy, Zhejiang Pharmaceutical College, Ningbo, China; ^5^ School of Pharmaceutical Sciences, Zhengzhou University, Zhengzhou, China

**Keywords:** iron, cancer, ferroptosis, ferritinophagy, prognostic, therapy

## Abstract

Iron is an essential trace element for the human body, and its deficiency or excess can induce a variety of biological processes. Plenty of evidences have shown that iron metabolism is closely related to the occurrence and development of tumors. In addition, iron plays an important role in cell death, which is very important for the development of potential strategies for tumor treatment. Here, we reviewed the latest research about iron metabolism disorders in various types of tumors, the functions and properties of iron in ferroptosis and ferritinophagy, and new opportunities for iron-based on treatment methods for tumors, providing more information regarding the prevention and treatment of tumors.

## Introduction

Iron (Fe) is one of the most abundant elements in the earth’s crust (1). The oxidation-reduction reaction mainly involves electron transfer between two chemicals. Iron, as a transition-metal, can exhibit a wide range of oxidation states, which makes it a multifunctional participant in redox reactions. Therefore, iron is an indispensable trace element to maintain life (2). This element plays a vital role in various cellular processes, such as cellular respiration (e.g., cytochrome c oxidase, ferredoxin, cytochrome, and Rieske protein), energy metabolism (e.g., aconitase, citrate synthase, succinate dehydrogenase, and isocitrate dehydrogenase), DNA replication, DNA synthesis and nucleic acid repair (e.g., the catalytic subunit of replicative DNA polymerases, DNA helicase and ribonucleotide reductase), and iron-dependent signaling (3, 4). Iron is also used in the synthesis of heme and iron–sulfur clusters (ISC), which are incorporated into proteins that carry out the citric acid cycle, oxidative phosphorylation, and many other essential functions (5, 6). However, although iron is essential for the normal physiological function of the human body, it may also be toxic in that it generates a large number of free radicals in the presence of hydrogen peroxide (7). For example, in the well-known Fenton reaction, ferrous iron (Fe^2+^) reacts with hydrogen peroxide to be oxidized to ferric iron (Fe^3+^) while generating hydroxyl radicals. When superoxide is present, the Fe^3+^ produced by the Fenton reaction can be reduced to Fe^2+^, and then the Fenton reaction will proceed again, which called Haber-Weiss reaction (8). Both the Fenton reaction and Haber-Weiss reaction can produce a large number of hydroxyl radicals. Hydroxyl radical is one of the most important oxidant found in human body, which can lead to peroxidation and apoptosis by attacking protein, lipids, nucleic acids, and carbohydrates (9, 10). In addition, excessive free radicals in the human body can lead to cell and tissue organ damage, and these processes are closely related to tumorigenesis.

Therefore, iron is not only an essential nutrient element, but also potentially toxic. Both aspects play important roles in the occurrence and development of tumors. This article mainly discusses iron metabolism disorders in tumors, ferroptosis, ferritinophagy, and the role of iron in tumor treatment.

## Physiological Mechanisms of Iron Regulation

**Figure 1:**
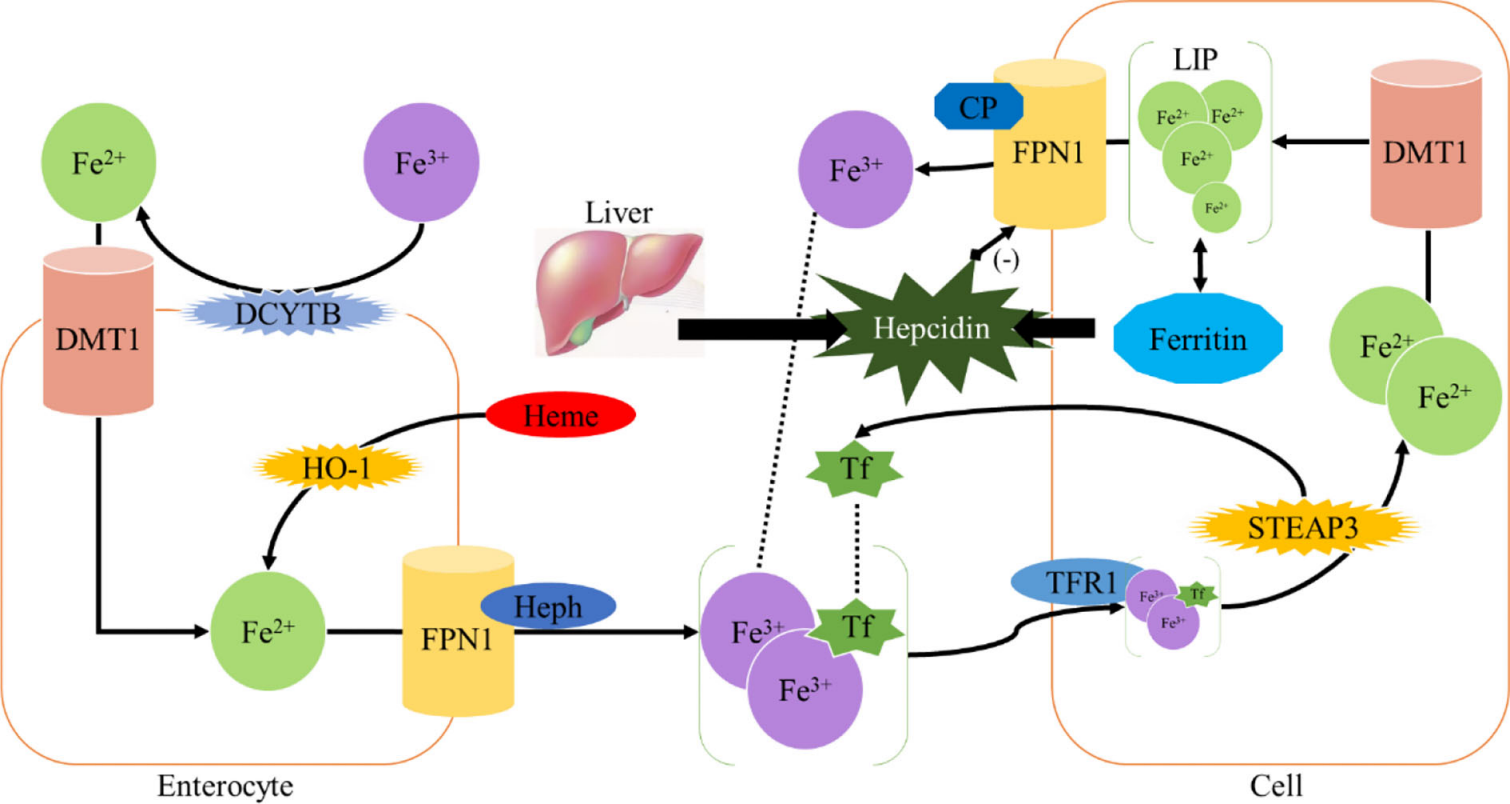
Iron metabolism.

Homeostasis of iron metabolism is a physiological process that needs to be strictly controlled. Iron is mainly present in the oxidized state (Fe^3+^) and is divided into dietary iron and environmental iron. Dietary iron primarily exists as either nonheme bound iron or heme (11). Heme iron has a higher absorption rather than nonheme bound iron. Iron in the diet is mainly reduced to Fe^2+^ in the duodenum by duodenal cytochrome B (DCYTB) and absorbed into intestinal epithelial cells under the synergistic effect of divalent metal transporter 1 (DMT1) (1). Heme iron is also absorbed by intestinal cells through an unknown mechanism and is metabolized by heme oxygenase-1 (HO-1) to release Fe^2+^. Heme iron is absorbed by intestinal cells through an unknown mechanism and degraded by heme oxygenase-1 (HO-1), releasing Fe^2+^, which is transported out of the cells by the iron efflux pump ferroportin (FPN1) on the basal side of the intestinal epithelial cells, and consequently oxidized into Fe^3+^ by hephaestin (HEPH) (1); then Fe^3+^ binds to transferrin (TF) and enters the circulation through the portal vein. Each transferrin in blood can bind two Fe^3+^ to form TF- [Fe^3+^]_2_ complex, which binds to transferrin receptor (TFR1) on the cell surface and is absorbed into cells to form endosome (12). Subsequently, it is reduced to Fe^2+^ by six-transmembrane prostate epithelial antigen 3 (STEAP3), which is then transported into cytoplasm by DMT1 to exert physiological functions or constitute the cytoplasmic labile iron pool (LIP) (1, 13). LIP can be taken up by non-hematopoietic cells causing parenchymal iron deposition which can result in free radical damage (14) (**Figure 1**).

**Figure 2:**
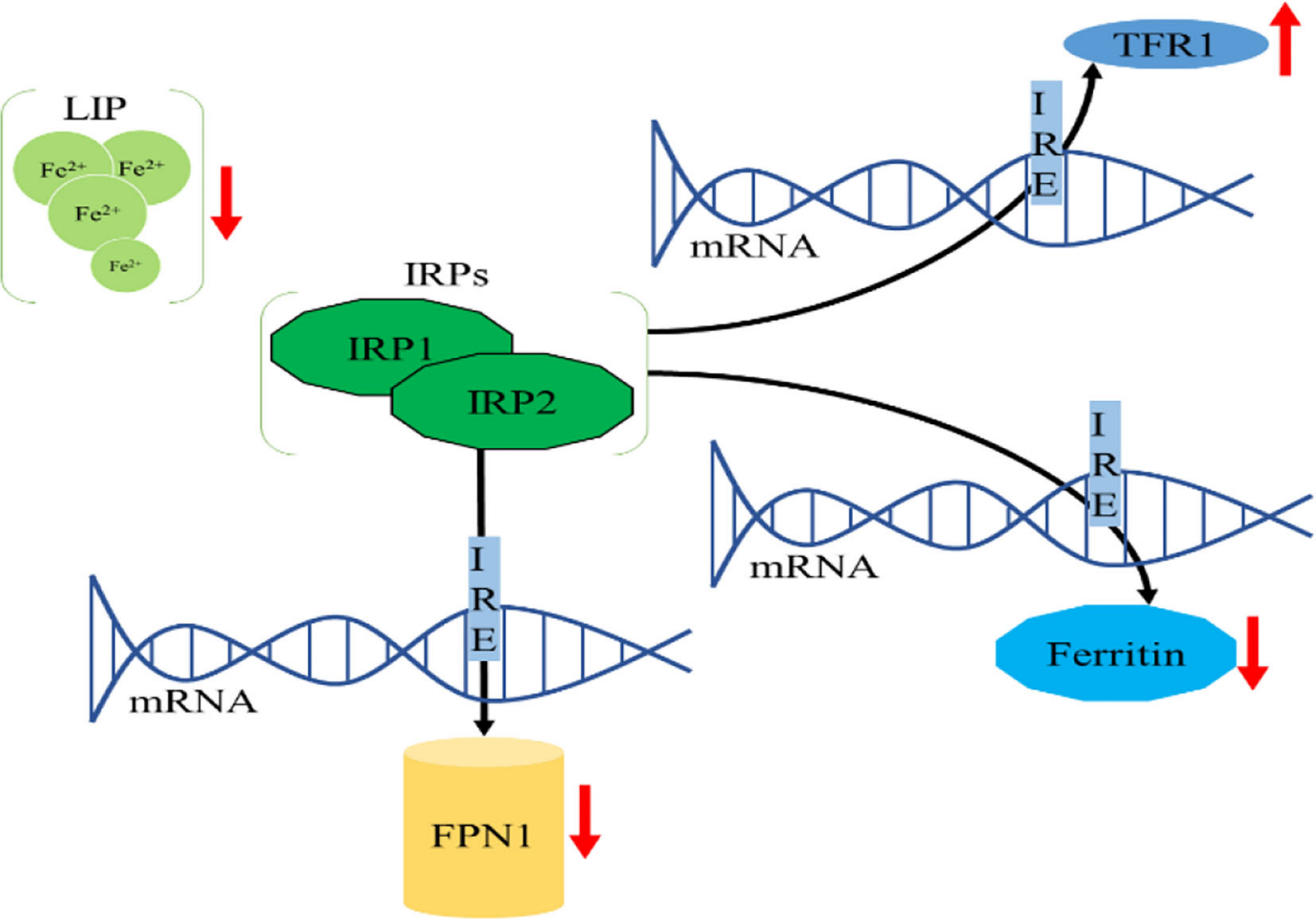
Cellular iron metabolism. **(A)** When the cellular iron levels are low, IRPs bind to the 5’ IREs of FPN1 and FT mRNA, resulting in translation repression. Additionally, IRPs bind to 3’ IREs in TFR1 mRNA to stabilize the DMT1 mRNA and increase TFR1 protein synthesis. These two effects can increase intracellular iron. **(B)** When the cellular iron levels are high, IRP1 converts to cytosolic aconitase and IRP2 is degraded, preventing IRP1 and IRP2 from binding to the IREs of these mRNAs. This permits unimpeded translation of FPN1 and FT and fosters degradation of DMT1 and TFR1 mRNA, leading to decreased cytosolic iron.

Intracellular iron homeostasis is mainly regulated through an iron-dependent protein network including iron-responsive element binding proteins (IRPs), in which both IRP1 and IRP2 are important components (15). It is noteworthy that thioredoxin family proteins are important mediators in iron metabolism since these proteins regulate the expression of IRPs (5). To ensure iron homeostasis, IRP binds to the corresponding iron responsive element (IRE) on the untranslated region of messenger RNA encoding the protein essential for cellular iron regulation (TFR1, DMT1), thereby participating in iron uptake (TFR1), storage (FT), redistribution, and efflux (FPN1) (2). In the absence of intracellular iron, IRP can inhibit the translation of FPN1 and FT, but increase the protein synthesis of TFR1. In contrast, when the intracellular iron is abundant, the synthesis of FPN1 and FT is increased due to the instability of IRP, while the degradation of TFR1 is promoted (16). IRP1/2 are key iron regulators for the maintenance of cellular iron homeostasis. IRP1 is cytosolic aconitase, an enzyme containing a [4Fe-4S] cluster. When absence of intracellular iron, there is insufficient iron for Fe-S biogenesis leaving an incomplete [3Fe-4S] cluster. The enzymatic activity of aconitase is lost and this protein then initiates its IRP activity, as IRP1. When the protein contains the [3Fe-4S] cluster it can bind to IREs (17, 18). Through these mechanisms, IRP can not only meet the metabolic needs of cellular iron, but also minimize the toxic effects of excessive iron (**Figure 2**).

The excess iron is mainly stored in liver, which also acts as an iron-sensing organ and controls systemic iron through the secretion of the peptide hormone hepcidin (19). Hepcidin is a 25-amino acid short peptide produced by the liver and encoded by the *HAMP* (20). FPN1, which is mainly expressed in the cell membrane and cytoplasm, is the only known 571-amino acid transmembrane protein in vertebrates that transports iron from intracellular to extracellular (21). When the intracellular or circulating iron level is relatively high, Hepcidin is generated from hepatocytes and secreted into the circulation system through a bone morphogenetic protein (BMP6) - mediated pathway (1, 22). Hepcidin can trigger the internalization and subsequent lysosomal degradation of FPN1 *via* binding to the basal side of the intestinal epithelium and to the FPN1 on the macrophage surface, preventing iron absorption from the digestive tract and iron circulation from the body, respectively (23). Conversely, the reduction of iron modulators causes FPN1 to absorb iron and increases systemic iron levels (24) (**Figure 1**). Therefore, FPN1 and ferritin are essential for maintaining iron homeostasis in the body.

## Iron Metabolism in Cancer

A large number of studies have shown that abnormal iron homeostasis is one of the markers of cancer (**Table 1**). As the metabolism and proliferation rate of tumor cells are generally higher than that of normal cells, so their demand for iron is also significantly higher than that of normal cells, this leading to the exceeding oxidative stress; however, tumor cells can exert a concomitant upregulation of antioxidant defenses for survival, such as activating antioxidant transcription factors and promoting the expression of various antioxidant genes (57). Conversely, since tumor cells are strongly dependent on iron for their growth/proliferation, they are more sensitive to iron depletion than normal cells. This imbalance in cancer is mainly manifested as increased iron metabolism, iron affinity, iron input, and inhibition of its output, thereby completing iron accumulation.

**Table 1:** Iron regulators in cancer.

Altered Player	Cancer	Regulation	Ref.
TFR1	glioblastoma	up	(25)
TFR1	leukemia	up	(26)
TFR1	breast cancer	up	(27,28)
TFR1	ovarian cancer	up	(29,30)
TFR1	hepatic carcinoma	up	(31,32)
TFR1	thyroid cancer	up	(33)
TFR1	colorectal cancer	up	(34–36)
DMT1	ovarian cancer	up	(30)
DMT1	pheochromocytoma	up	(37)
DMT1	colorectal cancer	up	(38)
STEAP3	glioblastoma	up	(39)
STEAP3	kidney renal clear cell carcinoma	up	(39)
STEAP3	Liver Hepatocellular Carcinoma	down	(28)
IRP2	colorectal cancer	up	(36)
IREB2	lung cancer	up	(40,41)
FPN	pancreatic cancer	down	(42)
FPN	multiple myeloma	down	(43)
FPN	thyroid cancer	down	(44)
Hepcidin	breast cancer	up	(45)
Hepcidin	colorectal cancer	up	(46)
Hepcidin	prostate cancer	up	(47,48)
Hepcidin	thyroid cancer	up	(44)
Hepcidin	breast cancer	up	(49)
Hepcidin	multiple myeloma	up	(50,51)
Hepcidin	non-Hodgkin’s lymphoma	up	(52)
Hepcidin	UUTUC	up	(53)
Hepcidin	Renal Cell Carcinoma	up	(53)
Hepcidin	hepatocellular carcinoma	down	(54)
*HAMP*	hepatocellular carcinoma	down	(55)
*HAMP*	cholangiocarcinoma	down	(56)

### Iron Uptake

#### TFR1

As early as 1980, studies have shown that *TFR1* is significantly up-regulated in breast cancer (27). Subsequent studies have confirmed that *TFR1* is highly expressed in various cancers such as glioma, leukemia, breast cancer, and ovarian cancer (25, 26, 28, 29). Recent studies have shown that Beclin 1 inhibits the proliferation of breast cancer cells by regulating the endocytosis transport and degradation of EGFR and TFR1 (58). Bajbouj et al. found that estrogen inhibited the synthesis of ferritin and enhanced the efflux of intracellular iron. Meanwhile, combining with doxorubicin, estrogen significantly reduced TFR1 expression and enhanced the sensitivity of breast cancer cells to doxorubicin (59). Using Gene Expression Profiling Interactive Analysis (GEPIA) database analysis, Xiao et al. found that TFR1 was significantly up-regulated in hepatocellular carcinoma tissues and hepatocellular carcinoma stem cells (31), and knocking down TFR1 reduced iron accumulation, reactive oxygen species (ROS) accumulation induced by erastin, and maintained mitochondrial function, thereby inhibiting tumor development (31). Additionally, a higher expression of *TFR1* is closely related to the activation of ERK signaling pathway in thyroid cancer, leading to the disorder of genes involved in abnormal accumulation of intracellular free iron and drug resistance (33).

Other studies have shown that targeting TFR1 can suppress tumor progression, such as MiR-107 can inhibit the proliferation, migration, and invasion of SW620 cells by targeting TFR1 (34); MiR-148a could reduce the proliferation of liver cancer cells by targeting TFR1 (32); EGFR regulates cellular iron homeostasis by regulating the redistribution of TFR1, and promotes the development of non-small cell lung cancer (60). Similarly, knock-down of *ST6GALNACIII* can down-regulate TFR1 and delay A549 cell proliferation (61). And Hui et al. found that TFR1 level was significantly up-regulated in non-small-cell lung cancer (NSCLC) by Fusaricide, which is a novel iron chelating agent and can efficiently inhibit the proliferation of a variety of human NSCLC cell lines (62). In addition, TFR1 is highly expressed in activated lymphocytes and malignant cells, and TFR1 antibody *ch128.1/IgG1* effectively inhibits the activation, growth, and immortalization of *EBV^+^* human B cells as well as the development of these cells into lymphoma-like tumors in immunodeficient mice (63). Chlorogenic acid inhibits the growth of pancreatic ductal adenocarcinoma cells by targeting the c-Myc-TFR1 axis, perhaps chlorogenic acid is a promising compound for pancreatic ductal adenocarcinoma therapy (64). However, although *TFR1* is highly expressed in colorectal cancer tissues, the authors demonstrated that down-regulation of *TFR1* promoted rectal cell migration and invasion through the JAK/STAT pathway (35). These results demonstrate that TFR1 might hold tumor-specific roles.

#### DMT1

DMT1 is highly expressed in various cancers, such as colorectal cancer and ovarian cancer (30, 38). Q. Wang et al. found that DMT1, TFR1, and ferritin were highly expressed in ovarian cancer cell spheres, and overexpression of DMT1 promoted the progression of ovarian tumor (30). In SDHB-Mutated Pheochromocytoma, iron accumulation caused by significant up-regulation of iron transport-related proteins, such as DMT1, TF, TFR2, can increase oxidative stress to some extent (37). In colorectal cancer, DMT1 can be induced through hypoxia-inducible factor 2α-dependent transcription (38). However, in hepatocellular carcinoma, patients with a lower expression of DMT1 displayed a worse disease-free survival, this effect was more significant in patients with advanced hepatocellular carcinoma (65). Notably, the carcinogenic activity of DMT1 is tightly correlated with its iron-transport activity, which is characterized by the evidence that tumor in DMT1-knocked out mice was weakened when being fed with a high-iron diet. Additionally, Desferoxamine (DFO) increases iron concentration by up-regulating the expression of DMT1 and TFR1, thereby promoting the migration of breast cancer cells (66). Similarly, the latest research by Chen et al. has revealed that the up-regulation of DMT1 and TFR1 is related to the activation of the IL-6/PI3K/AKT signaling pathway in triple-negative breast cancer cells, and enhance iron absorption (67). Consistently, targeting DMT1 can be used to potentially suppress tumor progression. For example, DMT1 inhibitors can selectively target stem cells in primary cancer cells and circulating tumor cells to inhibit the occurrence and development of tumors (68); Propofol, which is widely used in clinical practice for intraoperative general anesthesia and postoperative sedation, regulates DMT1 expression by modifying Ca2+-permeable α-amino-3-hydroxyl-5-methylisoxazole-4-propionic acid receptors (CPARs), thereby inhibiting tumor oxidative stress and glioma tumor growth (69).

#### STEAPs

The expression STEAPs also exhibits an upregulation in tumors and they can promote tumor cell proliferation as well as suppress apoptosis (70–73), for example, STEAP3 promotes glioma cell proliferation, invasion, and spheroid formation by inducing mesenchymal transformation, promoting TFR1 expression, and activating the STAT3-FoxM1 axis (39); In colorectal cancer, STEAP3 expression is significantly higher in tumor than that in colonic mucosa (74). However, they recently found that silencing lncRNA STEAP3-AS1(the antisense transcript of *STEAP3*) inhibited the proliferation, migration, and arrested colon cancer cells at the G0–G1 phase cancer cells through upregulation STEAP3 (75). Additionally, Z. Wang et al. found that among Burkitt’s lymphoma, the rarely studied iron reductase CYB561A3 was essential for Burkitt proliferation, but lymphoblastoid B cells with the EBV latency III program depended on STEAP3 iron reductase instead (76). Similarly, STEAP2 accelerates prostate cancer progression by promoting proliferation, migration, and invasion by regulating the transcriptional profiles of some genes involved in metastasis. And Burnell et al. proved that reducing the expression of STEAP2 inhibited the proliferation, migration, and invasion in prostate cancer cells (77).

### Intracellular Iron Regulation

IRP1 or IRP2 can increase intracellular iron and abnormal activation of them is closely related to many cancers. Compared with normal colonic mucosa, IRP2 is overexpressed in colorectal cancer, and is positively correlated with TFR1 expression. In addition, the expression of IRP2 is associated with mutations of BRAF, which primarily occur in the early stages of colorectal cancer and are often associated with poor prognosis (36). IRP1 and IRP2 are strictly regulated in tumors, and preferably modulate tumor progression in an iron-engaged signaling pathways, such as IRP2 is regulated by ubiquitin ligase FBXL5, which mediated IRP2 ubiquitination and degradation under the condition of sufficient iron; Dysregulation of FBXL5 has been associated with a poor prognosis of human hepatocellular carcinoma (78); A recent study demonstrated that FBXL5 is stabilized and increases iron levels when iron is at low levels, facilitating the production of [2Fe2S] cluster, which can serve as an FBXL5 cofactor, and by incorporating into the C-terminal LRR domain of FBXL5, and only when oxygen level is high enough to maintain the [2Fe2S] cluster in its oxidized [2Fe2S]^2+^ state could the SCF^FBXL5^ E3 ligase recruits IRP2 as a substrate for polyubiquination and degradation. This IRP2-FBXL5 interaction might help release IRP2 from IREs to alter the translation of iron metabolism genes (79). IRP2 is also regulated by the deubiquitinating enzyme OTUD1, which promotes TFR1-mediated iron transport *via* deubiquitinating and stabilizing IRP2, leading to increased ROS production, and the downregulation of OTUD1 has been found to be highly correlated with poor prognosis of colorectal cancer (80); A study linking a clinical trial of JBR.10 (n = 131) with a sample of patients from the University of Toronto Health Network (n = 181) indicated that the effect of the 15q25 mutation on lung cancer risk was associated with increased expression of *IREB2 (* 40); Another large-scale case-control study confirmed that the miRNA binding site SNP rs1062980 in the *IREB2* 3’ UTR might potentially alter *IREB2* expression to reduce the risk of lung cancer by regulating the binding of miR-29a (41). Additionally, the specific cause of dysregulation of IRP1/2 may be related to inhibition of TAp63 and activation of MDM2 (81, 82).Notably, chemotherapy and targeted-therapy may work together to disrupt IRP-mediated iron regulation, like Horniblow et al. found that the MEK inhibitor trimetinib consistently inhibited IRP2 expression in four colorectal cell lines, resulting in decreased TFR1 expression and increased ferritin expression (83); Miyazawa et al. found that cisplatin bound to Cys512 and Cys516 of human IRP2 and destroyed its function, based on which DFO combined with cisplatin resulted in increased iron consumption and reduced tumor growth in a mouse xenograft model of colonic adenocarcinoma (84); Yao et al. confirmed that erastin and RSL3 increased the expression of IRP1 and IRP2, knockdown of which conversely inhibited erastin- or RSL3-induce cell death, and IRP2 could enhance the promoting effect of IRP1 in melanoma cells (85).

### Iron Efflux in Cancer

#### Ferroportin

FPN1, the only iron export protein, is involved in the regulation of intracellular iron concentration, and its abnormal downregulation is also observed in most tumors (29, 47, 86, 87). The suppressive roles of FPN1 have been established in tumors, such as in prostate cancer cell lines, a low FPN1 level caused by up-regulation of ferritin promoted proliferation, migration, and apoptotic resistance, and overexpression of FPN1 induced p53 and autophagy, and reduced tumor growth *in vivo* (42); Overexpression of FPN1 decreased proliferation, colony formation, and tumor growth, as well as liver metastasis in breast cancer (88). In consistent, various studies have indicated that FPN1 regulates tumor progression *via* destroying iron homeostasis, as a study demonstrated that cadmium (Cd)-induced MDA-MB-231 cell proliferation, EMT, and migration were caused by inhibiting FPN1 expression and associated with destruction of iron homeostasis (89), and hepcidin secreted by thyroid cancer cells could decrease FPN1 and retain intracellular iron, thereby promoting cancer proliferation (44); another study showed that FPN1-mediated iron metabolism might play a role in chemosensitivity and treatment outcome of acute myeloid leukemia (90); Similarly, overexpression of ZNF217 promoted prostate cancer growth by inhibiting FPN1-conducted iron efflux (91); Xue et al. found that Nrf2, a transcription factor of *FPN1*, inhibited the proliferation and metastasis of prostate cancer cells by up-regulating FPN1, which was also related to the reduction of intracellular ferritin content (92), and Nrf2 was able to inhibit myeloma cell proliferation by promoting *FPN1* transcription (43). Additionally, Geng et al. demonstrated that knockdown of FPN1 accelerated erastin-induced suppression of neuroblastoma by increasing the accumulation of iron-dependent lipid ROS, and FPN1 inhibitors might provide a new approach for the chemosensitization of neuroblastoma (93); Tang et al. verified that USP35 could maintain the stability of its protein by deubiquitinating FPN1, and reduce the iron disorder triggered by erastin/RSL3, thereby promoting lung cancer cell growth and tumor progression. Meanwhile, knockdown of USP35 enhanced the sensitivity of lung cancer cells to cisplatin and paclitaxel by targeting FPN1 in lung cancer (94). However, a study showed that matrix/macrophage expression of Lcn-2 was associated with tumor onset, lung metastasis, and recurrence, whereas FPN1 was not by analyzing the expression profiles of *lipocalin-2* (*Lcn-2*) and *FPN1* through a model of T-oncogene (PyMT) breast cancer in spontaneous polymerases and mining publicly available TCGA and GEO database from gene expression synthesis (95). Macrophages provide iron for the microenvironment of breast tumors *via* forcing secretion of Lcn-2-bound iron and increasing expression of FPN1 (95). Nevertheless, the reason why the matrix/macrophage expression of FPN1 is not associated with tumor onset, lung metastasis, and recurrence requires further investigation.

#### Hepcidin

Disorders of iron modulators in cancer cause changes in iron homeostasis. Hepcidin, a negative regulator of FPN1, is significantly up-regulated in various tumors, such as breast, colorectal, and prostate cancer (45–47). And numerous studies have shown the promoting effects of hepcidin on tumor progression, for example, Schwartz et al. found that compared with wild-type littermates, mice lacking hepcidin in colonic tumor epithelium significantly reduced the number, tumor burden and size in the sporadic model of colorectal cancer, whereas lacking of FPN1 led to intracellular iron accumulation and promoted tumor occurrence (96); Lopes et al. found that acute myeloid leukemia had a unique iron component, mainly manifested as a low expression of transferrin, and high expression of ferritin and hepcidin; notably, these characteristics were not related to inflammation or blood transfusion (50); Zhao et al. confirmed that hepcidin enhanced the proliferation, migration, and anti-apoptotic capabilities of prostate cancer cells by reducing the expression of FPN1 and increasing intracellular iron level (48); Additionally, Zhou et al. identified that the synthesis of hepcidin is regulated by SOSTDC1, the BMP4/7 antagonist, providing a new mechanism for cellular iron dysfunction *via* the E4BP4/G9a/SOSTDC1/hepcidin pathway in the thyroid gland, which can inhibit the secretion of hepcidin and proliferation of thyroid cancer cells (44).

In addition to the iron modulators synthesized by cancer cells, systemic iron disorders in cancer patients can also be observed (88, 97, 98). Serum hepcidin is elevated in many cancer patients, including prostate cancer, breast cancer, multiple myeloma, and non-Hodgkin’s lymphoma (48, 49, 51, 52).A study of 456 cases of primary gastric adenocarcinoma and 900 matched controls with an average of 11 years of follow-up showed a significant negative correlation between gastric cancer risk and serum hepcidin level, which was mainly caused by ferritin level (99). Additionally, treatment with morotinib was found to decrease serum hepcidin and improve iron metabolism and erythropoiesis in a Phase 2 clinical trial of morotinib for bone marrow fibrosis (100). Similarly, a retrospective study, including 38 patients suffering from upper urinary tract urothelial carcinoma (UUTUC), 94 patients Suffering from Renal Cell Carcinoma (RCC), and 21 patients without infections or cancer, showed that serum hepcidin level was significant increased compared to sera of controls in patients with UUTUC and RCC, and high serum hepcidin was associated with cancer recurrence and metastasis (53).

Interestingly, liver cancers showed a drastic reduction of hepcidin expression compared to benign liver tissues (101, 102). Furthermore, in patients of the White race with no history of alcohol consumption, down-regulation of hepcidin is associated with rapid cancer progression and poor disease-specific survival. Hepcidin expression is positively correlated with BMP6/interleukin -6 (IL6) cytokines and cytotoxic immune infiltration in liver cancer tissues (54). In addition, blocking hepcidin with its antagonist furthiomine could moderately reduce sorafenib-induced apoptosis in HepG2 and Huh7 cells (54). In consistent, *HAMP*, the coding gene for hepcidin is mainly expressed in benign liver tissues but significantly reduced in hepatocellular carcinoma tissues (54, 55). Similarly, Z. Wang et al. found that *HAMP* was decreased and associated with the chemokine CCL16 in Cholangiocarcinoma (CHOL), the second common malignant tumor in the liver, indicating that *HAMP* may contribute to the immune activation in CHOL microenvironment (56).

Heme has been indicated to promote cell proliferation in leukemia and lung cancer, and increase HO-1 activity promotes invasion and migration of breast cancer cells (103–105). Up-regulation of BMP, which induces cancer cells to secrete hepcidin, has also been observed in various tumors, including breast, prostate, and bladder cancers (47, 106, 107).

In conclusion, cancer cells usually increase the input of iron and inhibit its output, thereby achieving iron accumulation. However, it is not entirely clear how they respond to the increased instability.

## Iron and Cell Death

### Iron and Ferroptosis

**Figure 3:**
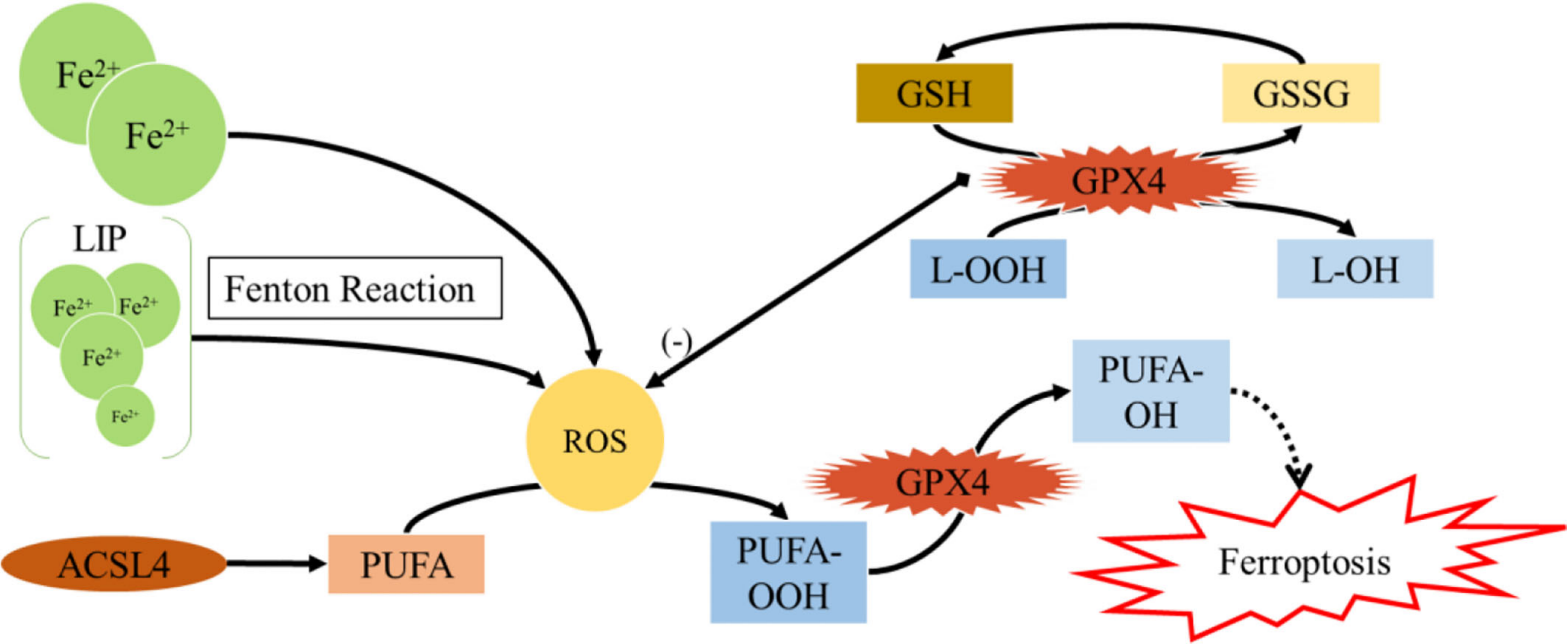
Ferroptosis.

In recent years, non-apoptotic cell death has attracted a widespread attention in tumor therapy, among which ferroptosis is defined as iron-dependent regulatory necrosis caused by membrane damage mediated by massive lipid peroxidation (108–110) (**Figure 3**). Upon being experienced ferroptosis, cells show unique signs such as cell membrane rupture, cytoplasmic swelling, cytoplasmic organelle swelling, mitochondrial membrane density increase, mitochondrial cristae reduction/disappearance, mitochondrial outer membrane rupture, *etc.* Ferroptosis can occur through two main pathways: the external or transporter-dependent pathway, and the internal or enzyme-regulated pathway (111). Ferroptosis is caused by the redox imbalance between the production of oxidants and antioxidants, which is driven by the abnormal expression and activity of numerous redox active enzymes that produce or detoxify free radicals and lipid oxidation products (109, 111).

As the center of metabolism, mitochondria are an important source of ROS in most mammalian cells. Earlier studies showed that mitochondrial-mediated ROS production was not necessary for ferroptosis (108). However, recent studies have shown that mitochondrial-mediated ROS production, DNA stress, and metabolic reprogramming are necessary for lipid peroxidation and induction of ferroptosis (112–114). Readers can obtain a more comprehensive understanding of ferroptosis by referring to other relevant documents (111, 115–118). Here we focus on recent research, aiming to clarify the possible novel mechanisms of ferroptosis in cancer.

The previous study suggested that the stability of IRP2 protein was mainly regulated by E3 ubiquitin ligase FBXL5 (78), while Terzi et al. confirmed a new regulatory mechanism of IRP2, which senses the absence of ISC synthesis, and ISC defects could enhance the binding of IRP2 to target mRNA ignoring the changes in IRP1, IRP2, and FBXL5 protein levels. ISC are made up of iron and sulfur ions to form [1Fe-0S], [2Fe-2S], [3Fe-4S] and [4Fe-4S] clusters (6, 119). Suppressing ISC synthesis can activate IRP2 and promote ferroptosis sensitivity (120); in line with this finding, insufficient ISC maintenance has been shown to robustly activate the iron-starvation response and trigger ferroptosis (121). Notably, inorganic sulfur is first produced from the cysteine by the cysteine desulfurase NFS1 and ISC is formed on the ISC assembly enzyme (ISCU) with the help of frataxin (FXN) (122),Which was indicated to be localized in the mitochondrial matrix and participated in the biosynthesis of ISC and FXN deficiency accelerates erastin-activated ferroptosis (123). Furthermore, overexpression of ISCU significantly attenuated Dihydroartemisinin-induced ferroptosis by regulating iron metabolism, rescuing the mitochondrial function and increasing the level of GSH (124). Additionally, FXN could activate NFS1 and accelerate a rate-limiting sulfur transfer step of ISC assembly, and suppression of NFS1 make cancer cell be sensitive to ferroptosis (121, 125). Besides, Chafe et al. identified a novel synthetic lethal interaction between carbonic anhydrase IX (CAIX) and NFS1 by elucidating the important role of CAIX in redox homeostasis and the prevention of ferroptosis through pH regulation, which may facilitate researchers to develop new strategies for the treatment of solid tumors (126).

The latest research provides more possibilities that targeting ferroptosis may be a new strategy for tumor treatment. For example, Mao et al. identified a ferroptosis-defensive mechanism mediated by Dihydroorotate dehydrogenase (DHODH) in the mitochondria, which works with mitochondrial glutathione peroxidase 4 (GPX4) to reduce ubiquinone to panthenol, thereby inhibiting ferroptosis in mitochondrial inner membrane, while Brequinar (a DHODH inhibitor) selectively inhibits the proliferation of tumor cells with low GPX4 expression by inducing ferroptosis, thus it can occur synergistically to induce ferroptosis and inhibit the growth of tumor cells with high GPX4 expression by the combined use of Brequinar and sulfasalazine (ferroptosis inducers) (127); Subsequently, Ding et al. demonstrated that DMOCPTL induced ferroptosis and apoptosis primarily through GPX4 ubiquitination in triple-negative breast cancer cells (128); Additionally, D. Chen et al. confirmed that IPLA2β inhibited ferroptosis by cleaving through lipid peroxide for detoxify without depending on GPX4, and that the absence of iPLA2β had no significant effect on the normal development or cell viability of normal tissues, thus, iPLA2β may become a new target for ferroptosis-targeted therapy for tumor (129); Furthermore, X. Wang et al. demonstrated that SOX2 promoted *SLC7A11* transcription by binding to *SLC7A11* promoter, and oxidation at *Cys265* of SOX2 inhibited its activity and decreased the self-renewal capacity of lung cancer stem cell-like cells. This suggests that oxidation of SOX2 could be a potential target for ferroptosis-targeted treatment for cancer (130). Moreover, as cell density-dependent E-cadherin and Merlin/Neurofibromin (NF2) loss can induce ferroptosis, Bao et al. found that NF2-inactivated meningioma cells were sensitive to Erastin-induced ferroptosis by analyzing 35 meningioma samples (10 *NF2* loss and 25 *NF2* wildtype), and further confirmed that myoenhancer factor 2C (*MEF2C)* acted as a promoter of *NF2* and *CDH1*, thereby inhibiting ferroptosis-related lipid peroxidation and meningioma cell death (131). Notably, Kremer et al. indicated that aspartate aminotransferase (GOT1) could damage mitochondrial oxidative phosphorylation and promote catabolism, resulting in the increase of unstable iron pool and susceptibility to ferroptosis, this effect suggests that inhibiting GOT1 could destroy the redox balance and proliferation in pancreatic ductal carcinoma, and establishes a biochemical link between GOT1 and ferroptosis (132). Few studies reported the direct crosstalk between ferroptosis and antitumor immunity, until Wang et al. reported that CD8^+^ T cells induce ferroptosis in tumor cells, which is the direct evidence of the connection between ferroptosis and antitumor immunity (133). They found that interferon gamma (IFNγ) released from CD8^+^ T cells downregulated the expression of SLC3A2 and SLC7A11, impaired the uptake of cystine by tumour cells, and promoted ferroptosis. Thus, T cell-promoted tumour ferroptosis is an anti-tumour mechanism, and targeting this pathway in combination with checkpoint blockade is a potential therapeutic approach.

Furthermore, studies also demonstrated that activating ferroptosis and apoptosis immensely increased chemotherapy sensitivity, which might provide strategies for the combination therapy for cancers. For example, Ye et al. found that the synergy of apoptotic activator and ferroptosis inducer could significantly enhance the cytotoxic effect of gemcitabine in pancreatic cancer, providing a new strategy for pancreatic cancer treatment (134); Hong et al. unveiled a novel treatment strategy for ovarian cancer through a combined use of Poly (ADP-ribose) polymerase S (PARP) inhibitor and ferroptosis inducer (135); Sun et al. identified that QSOX1 (Quiescin sulfhydryl oxidase 1) enhanced sorafenib-induced ferroptosis by promoting the ubiquitination degradation of EGFR and inhibiting EGFR activation and thus inhibiting NRF2, providing QSOX1 as a new candidate target for a sorafenib-based combination therapy in advanced hepatocellular carcinoma or EGFR-dependent tumor types (136).

There are also some new discoveries focusing on the relationship between lipid peroxidation and ferroptosis. Tan et al. found a hypoxia-inducible factor-dependent adipokine chemerin, which could prevent fatty acid oxidation and lead to escape from ferroptosis, and targeting chemerin reduced lipid storage and tumor growth (137); Lang et al. demonstrated that supplementing unsaturated fatty acids while inhibiting the biogenesis of lipid droplets could induce ferroptosis in acidic cancer cells (138); Additionally, Dierge et al. found that a diet rich in n-3 long-chain unsaturated fatty acids significantly inhibited tumor growth in mice, indicating that supplementation of dietary unsaturated fatty acids can serve as a selective adjuvant anti-tumor approach (139); Furthermore, supplementation of dietary unsaturated fatty acids may serve as a selective adjuvant anti-tumor approach. W. Liu et al. verified that dyslipidemia affected the occurrence and development of tumors by selectively resisting ferroptosis, which primarily due to the continued expression of GPX4 in the metabolism of 27- hydroxycholesterol-resistant cells (140); In consistent, Beatty et al. identified conjugated linoleic acids, including α-eleostearic acid (αESA), acted as inducers of ferroptosisby acyl coenzyme a synthetase long-chain isomer 1 and interfering with the biosynthesis of triglycerides inhibited αESA-induced ferroptosis, but not GPX4-inhibited ferroptosis (141).

Notably, many extracts from plants and herbs also exhibit anti-tumor effects by inducing ferroptosis. Z.X.Wang et al. found that quercetin could promote the degradation of lysosomal-dependent ferritin and the release of free iron, this effect and quercetin-induced ROS production synergistically led to lipid peroxidation and ferroptosis (142). C.Y. Wu et al. found that *Dihydroisotanshinone I* (*Radix Salviae Miltiorrhizae* extract) could induce ferroptosis in lung cancer cells by blocking the expression of GPX4 protein (143). And Wen et al. confirmed that 18-β-glycyrrhetinic acid could promote the production of ROS and RNS by activating NADPH oxidase and iNOS, and reducing GSH and GPX activities in triple-negative breast cancer cells, thereby accelerating lipid peroxidation and leading to ferroptosis (144).

### Iron and Ferritinophagy

**Figure 4:**
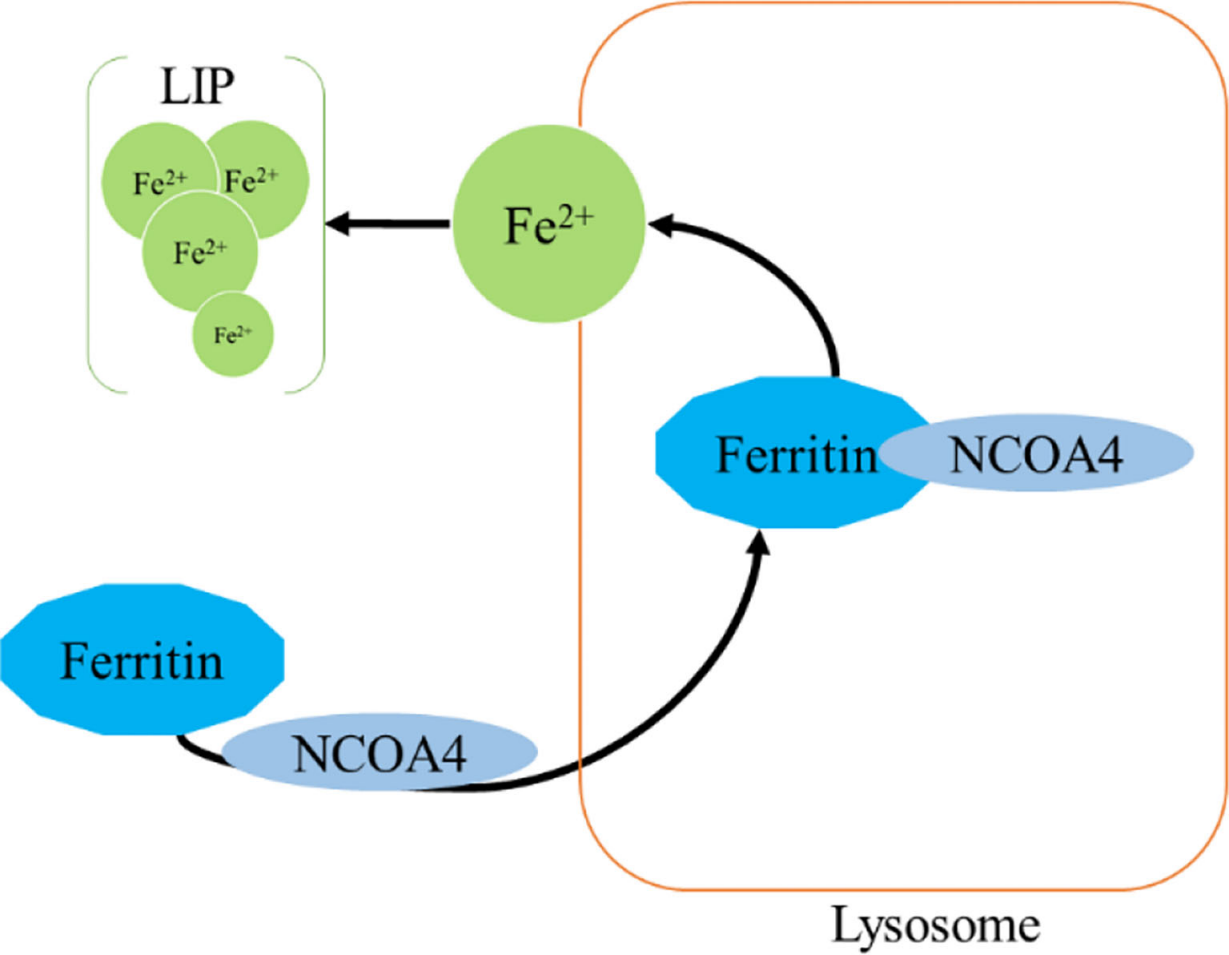
Ferritinophagy.

Ferritinophagy is a type of cell selective autophagy, in which the ferritin (mainly ferritin heavy chain 1) is degraded in autophagosomes mediated by nuclear receptor coactivator 4 (NCOA4), leading to the ferritin-bound iron to be released as free iron (145, 146) (**Figure 4**). Ferritinophagy contributes to the initiation of ferroptosis through degradation of ferritin, which triggers labile iron overload (IO), lipid peroxidation, membrane damage, and cell death (147), and plays a certain role in tumorigenesis.

Ferritin is a complex that can hold 4,500 Fe^3+^ and is widely present in mammalian cells. The ferritin complex is assembled by the heavy chain (*ferritin heavy chain 1, FTH1*) and the light chain *(ferritin light chain, FTL*) (148). *FTH1* has a ferrous oxidase activity and can catalyze the oxidation of Fe^2+^ to Fe^3+^, thereby reducing a large number of Fe^2+^-produced free radicals participating in the Fenton reaction, and the damage of free radicals to tissues and organs (149, 150). Iron combines with each other through ferritin iron pores and Fe^2+^ is further oxidized to Fe^3+^ by *FTH1* in the ferritin cage, resulting in the inert deposition of Fe^3+^, which cannot use or generate ROS in cells (151).The main way of ferritin to release iron is selective autophagy mediated by NCOA4, which binds to ferritin and is transported to lysosomes, where ferritin is degraded and iron is released for cell use (145). NCOA4 selectively interacts with *FTH1* subunit of ferritin through its conserved C-terminal domain and key residues on *FTH1* (152). Notably, NCOA4 is regulated by HECT and the RLD domain containing E3 ubiquitin protein ligase2 (HERC2) (153).

Mechanistically, when cellular iron level is high, NCOA4 and HERC2 have an iron-dependent effect, resulting in the degradation of NCOA4 through ubiquitin proteasome system, thereby reducing the release of iron and facilitating the storage of ferritin. On the contrary, under low cellular iron conditions, the interaction between NCOA4 and HERC2 is reduced, leading to an increase of NCOA4 level, which increases the degradation of ferritin and iron autophagy flux to supplement cellular iron (154, 155). Thus, a proper amount of ferritinophagy can maintain the balance of iron in cells. However, an excessive activation of ferritinophagy can lead to disease induced by iron overload. Importantly, studies have found that there is a close link between iron release caused by ferritinophagy and ROS damage in ferroptosis (115, 156, 157). Under certain conditions, iron release caused by ferritinophagy is a component of ferroptosis, and may be a direct driving factor of ferroptosis (115, 156, 157). The role of ferritinophagy-related genes in cancer progression has been confirmed (158–164). Here, we focus on clarifying the new discovers of NCOA4 and *FTH1* in cancer.

The expression of NCOA4 is lower in Clear cell renal cell carcinoma (ccRCC) tissues compared with normal tissues, and low NCOA4 expression is closely related to high-grade malignant tumors and advanced TNM staging (165, 166). Some studies have indicated the critical role of NCOA4-mediated ferritinophagy in tumor progression, such as knockdown of *COPZ1* leads to an increase in NCOA4, resulting in the degradation of ferritin, and ultimately ferroptosis in glioblastoma (167); Vara-Pérez et al. found that melanoma cells lacking BNIP3 showed increased intracellular iron levels caused by NCOA4-mediated increase in ferritinophagy (168). Nevertheless, NCOA4-mediated ferritinophagy is not effective in all tumors as Hasan et al. found that the destruction of ferritinophagy by NCOA4 knockout resulted in minor differences in growth under basal and iron-restricted conditions in colon cancer cells; Additionally, NCOA4 does not engage in cell death induced by 5-fluorouracil and erastin (169).

Furthermore, *FTH1* is also another important gene involved in ferritinophagy. Hayashima et al. demonstrated that in glioblastoma cells, cystine deprivation rather than L-buthionine sulfoximine treatment caused ferroptosis, although they both lead to depletion of cellular GSH, and NCOA4-mediated degradation of *FTH1* was observed in cystine-deprived cells but not in L-buthionine sulfoximine -treated cells. Besides, inhibition of *FTH1* degradation suppressed cystine deprivation-induced ferroptosis. This suggests that cystine deprivation-induced ferroptosis required not only GSH depletion, but also intracellular iron accumulation, and ferritinophagy plays an essential role in cystine deprivation-induced ferroptosis (170). Notably, due to insufficient *FTH1*, Erastin or RSL3 induces ferroptosis in neuroblastoma N2A cells, but fails to induce normal nerve cell death, indicating that ferroptosis may be a promising therapeutic target for neuroblastoma (171).

## Iron in Cancer Therapeutics

### Iron in Cancer Prognosis

Because iron is essential at all stages of tumor development, survival, proliferation, and metastasis, we analyzed the prognostic value of iron and iron regulatory genes. It has shown that serum ferritin has prognostic value in various cancers, and elevated serum FT levels are associated with poor prognosis (46, 172–175). However, the increase of serum ferritin are not necessarily associated with upregulated iron level in the body, this means that iron level is regulated by other stimuli, such as inflammation (2). Therefore, we hereby summarize the association between factors related to iron metabolism and prognosis (**Table 2**).

**Table 2:** Iron regulators in cancer prognosis.

Altered Player	Cancer	Regulation	prognosis	Ref.
TFR1	hepatic carcinoma	up	poor	(176,177)
TFR1	ovarian cancer	up	poor	(29)
TFR1	lung cancer	up	poor	(178)
TFR1	gastric cancer	up	poor	(179)
TFR1	glioblastoma	up	poor	(180)
TFR2	glioblastoma	up	good	(181)
TFR1	colorectal cancer	up	good	(182)
FPN1	breast cancer	down	poor	(45)
FPN1	lung cancer	down	poor	(178)
FPN1	adrenocortical carcinoma	down	poor	(183)
FPN1	multiple myeloma	down	poor	(43)
FPN1	clear cell renal cell carcinoma	down	poor	(184)
STEAP3	glioblastoma	up	poor	(39)
STEAP1	breast cancer	up	good	(185)
STEAP2	breast cancer	up	good	(185)
STEAP4	breast cancer	up	good	(185)
Hepcidin	breast cancer	up	poor	(186)
FTL	ovarian cancer	up	poor	(187)
DMT1	ovarian cancer	up	poor	(187)
*HAMP*	ovarian cancer	up	poor	(187)
DMT1	hepatocellular carcinoma	low	poor	(65)

In view of the important role of TFR1 in iron uptake, TFR1 has been identified as a prognostic marker for many tumors, such as TFR1 expression in ER^-^ tissue was significantly higher than that in ER^+^ tissue (188); Compared with adjacent non-cancer tissues, the mRNA expression level of *TFR1* in hepatocellular carcinoma tissues is remarkably increased, and the expression of *TFR1* and *TFR2* is negatively correlated with tumor differentiation (176, 177); Furthermore, patients with a high TFR1 expression have a high risk of recurrence and death after hepatectomy (177); Similarly, TFR1 is significantly overexpressed in ovarian cancer and glioblastoma, and TFR1 expression may be related to tumor staging, progression or the short survival time (29, 180). Additionally, the activation of EGFR induces cell redistribution of TFR1, and the highly expressed TFR1 is closely related to the progression of lung cancer (178). The assessment of 178 gastric cancer tissues revealed a negative correlation between TFR1 expression and patient prognosis, and the TFR1 positive sorting cells showed a strong proliferative capacity; however, the TFR1-negative cells showed a more aggressive tumor features (179). In contrast, although TFR2 is also significantly up-regulated in glioblastoma, but its up-regulation predicts a good prognosis for glioblastoma (181). Notably, in colorectal cancer, patients with a low expression of TFR1 have a shorter survival compared with patients with positive TFR1 expression (182). These results suggest that the roles of TFR1/2 in tumor prognosis might be tumor-specific.

Similarly, *FPN1* has been identified as a favorable prognostic marker for many tumors (189). In a gene expression profile of approximately 800 breast cancer patients, it was reported that decreased *FPN1* expression was significantly associated with reduced metastasis-free and disease-free survival (45). And *FPN1* mRNA and protein expression are significantly down-regulated, and patients with a low *FPN1* expression have a poor prognosis in lung cancer and adrenocortical carcinoma (ACC) (178, 183). Additionally, a similar effect was observed in multiple myeloma and ccRCC (43, 184). Furthermore, STEAP3 is highly expressed in glioblastoma and is closely related to the decreased overall survival rate (39); however, a survival analysis showed that breast cancer patients with high levels of STEAP1, STEAP2, and STEAP4 had an overall good prognosis (185).

Higher hepcidin is also reported to be associated with a shorter recurrence time of distant breast cancer, and hepcidin may be associated with a poor prognosis for breast cancer in obese women (186). Survival analysis showed that ovarian cancer patients with a high expression of *FTL*, DMT1 and *HAMP* showed a poor overall survival rate (190). IL-6 and BMP control hepcidin secretion in cancer and IL-6 level is elevated in lung cancer patients with poor prognosis (187).

The progression of breast cancer is negatively correlated with hemoglobin (Hb) and positively correlated with ferritin levels (191). Compared with the high-DMT1 group, liver cancer patients with a low DMT1 expression have a worse disease-free survival rate, which is particularly obvious in patients with advanced liver cancer (65).

### Iron in Cancer Therapy

Many approaches have been constructed to treat cancer against intracellular iron metabolism disorders: one strategy is to deplete iron of tumor cells, such as iron chelator; another important one is to generate cytotoxic level of ROS or ferroptosis through excess iron in tumor cells (**Table 3**).

**Table 3:** Iron in Cancer Therapy.

Name	Mechanism	Ref.
Cyst(e)inase	degrading cysteine	(133,192,193)
DFO	iron depletion	(194)
DFO	iron depletion	(195)
Triapine	iron depletion	(196)
Dp44mT	iron depletion	
Fusaricide	iron depletion	(62)
Ga	iron depletion	(197)
Tris (8-quinolinolato) Gallium (III) (KP46)	iron depletion	(198)
Gallium Mallotate	iron depletion	(198)
Ferrocene derivatives	elevated iron levels	(199)
Ferumoxytol	elevated iron levels	(200)
ascorbic acid	ferroptosis	(201–205)
salinomycin derivatives	ferroptosis inducers	(206)
DMT1 inhibitor	target DMT1	(68)
HFn-PTX	target TFR1	(207)
DOX-FTH	target TFR1	(179,208)
miR-375	target *SLC7A11*	(209)
miR-148a	targeting *TFR1*	(32)
miR-184	targeting *HAMP*	(210)
miR-200b	targeting ferritin	(211)

#### Iron Depletion

Iron chelators can change the metabolism of tumor cells by reducing the intake of iron. It has also been demonstrated that iron chelators inhibit ribonucleotide reductase activity and play important roles in various signaling pathways associated with tumor progression and metastasis (212, 213). In a study of patients with advanced hepatocellular carcinoma, 20% of patients responded to treatment with DFO (194); In another study of nine patients with neuroblastoma, seven patients had reduced bone marrow infiltration after DFO treatment (195). However, iron chelators appear to be effective only in specific tumors. Other studies have shown that DFO is ineffective in the treatment of hormone-reducing refractory prostate cancer and recurrent neuroblastoma (214, 215). Notably, other chelating agents, such as Triapine and Dp44mT, have entered clinical experiments (216, 217). After receiving Triapine treatment, 76% of patients with advanced hematological malignancies had a 50% reduction in white blood cell count (196). However, Triapine only showed the smallest overall response rate in metastatic renal cell carcinoma and recurrent and metastatic head and neck squamous cell carcinoma (218, 219). Additionally, Hui et al. confirmed that Fusaricide, a novel iron chelating agent, could induce apoptosis by activating Caspase-3 (62). Furthermore, iron chelators have been shown to alter macrophage polarization, and immune signaling. Prill et al. showed that DFO administration led to high iron efflux by decreasing ferritin expression in the tumor-associated macrophages (220). EC1 (a thiosemicarbazone chelator) treatment reversed the positive effect of macrophage-conditioned media on the proliferation and migration of cancer cells (221). Moreover, the safety and efficacy of iron chelators as cancer therapeutics have been tested in clinical trials, gastrointestinal symptoms and fatigue appear to be the most prevalent toxicities (194, 219, 222, 223).

The use of iron chelators in combination therapy has also been partially investigated. DFO was combined with many different chemotherapeutic drugs, such as cyclophosphamide, etoposide, cisplatin, carboplatin, and thiotepa (190, 224, 225). In a cohort of patients with advanced neuroblastoma and primitive neuroectodermal tumors, DFO was effective in combination with cyclophosphamide, etoposide, carboplatin, and thiotepa (224). In another study of 37 patients with accelerated myeloproliferative tumors and secondary acute myeloid leukemia, the combination of Triapine and fludarabine (a DNA synthesis inhibitor) showed 49% overall response rate and 24% complete response rate (226).

Gallium salts, which belong to the group IIIa metals and have common chemical properties with iron, are variants of the iron depletion strategy in tumor therapy. Therefore, gallium (Ga) is used to disrupt iron metabolism to mimic the behavior of iron. Gallium can be incorporated into protein and enzymes that use iron as a cofactor, such as ribonucleotide reductase, which inactivates enzymes that require iron to function and lead to an increase in mitochondrial reactive oxygen species (197). Thus, the gallium-based compound exhibits antitumor activity by disrupting iron-dependent tumor metabolism. The spectrum antineoplastic activity of gallium nitrate in the clinic was evaluated in Phase II clinical trials. Among a number of cancers examined, gallium nitrate displayed promising antineoplastic activity in patients with nonHodgkin’s lymphoma and bladder cancer (198, 227–229).The anti-cancer activity of newer gallium-ligands have been developed and are being undergone clinical evaluation, such as Tris (8-quinolinolato) Gallium (III) (KP46) and Gallium Mallotate, which may be more effective than the nitrate salt used in the original clinical formulation of gallium (198, 230).

#### Elevated Iron Levels

The strategy in contrast to iron depletion is to supply cells with excess iron. Excess iron combines with high levels of unstable iron in tumor cells to produce large amounts of ROS to eliminate tumor cells. For example, the metal-containing drugs Ferrocene derivatives are stable and exhibit favorable redox properties, inhibiting proliferative activity of tumor cell lines (199). Additionally, Ferumoxytol is an iron oxide nanoparticle approved by the FDA for the treatment of clinical iron deficiency, and studies have shown that ferumoxytol can produce excessive amounts of free iron, the reactive oxygen species produced by which can cause cell death, increase oxidative stress, and reduce tumor burden cells in mouse leukemia models and patients (200).

What’s more, ascorbic acid therapy is a variant of cancer treatment strategy by affecting the oxidation state of iron and increasing LIP levels, which is indicated for various tumors (201–203). Multiple clinical trials of this therapy are currently being pursued. Such as NCT02344355, NCT02420314, NCT02905578, NCT02905591, NCT03508726, NCT03602235, and NCT03799094 (https://clinicaltrials.gov/) (204, 205). However, it must be noted that if a large amount of divalent iron is oxidized to trivalent iron, you will suffer from methemoglobinemia. Furthermore, high levels of iron in the blood reduce our healthy years of life, and keeping these levels in check could prevent age-related damage (231).

#### Ferroptosis Inducers

The ferroptosis inducers can target the treatment of tumor by increasing the iron level of the tumor cells. Conversely, targeting ferroptosis inhibitors also have been shown to suppress tumor progression, for example, depletion of cystine or cystine by cyst(e)inase (an engineered enzyme that degrades both cystine and cysteine) in combination with checkpoint blockade synergistically enhances T cell-mediated anti-tumour immunity and induces ferroptosis in tumor cells (133); Cyst(e)inase also can synergize with thioredoxin reductase inhibition for suppression of pancreatic tumor growth (192); Additionally, administration of cyst(e)inase induces tumor-selective ferroptosis and inhibited pancreatic tumor growth (193). IFN-γ derived from immunotherapy-activated CD8^+^ T cells synergizing with radiotherapy-activated ataxia-telangiectasia mutated (ATM) suppresses SLC7A11, to induce cystine uptake, enhance tumor lipid oxidation and ferroptosis in human fibrosarcoma and melanoma cells (232). Notably, as we and others previously showed that that ferroptosis inducers target tumor stem cells to inhibit tumor proliferation and reduce metastasis (205, 233, 234). Several US Food and Drug Administration (FDA)-approved drugs have been shown to induce ferroptosis in tumor cells in preclinical models, but the clinical utility of these ferroptosis inducers require further investigation (2). Another study has shown that salinomycin derivatives can trigger the ferroptosis pathway in cancer stem cells (206). Furthermore, Andreea L Turcu et al. developed a DMT1 inhibitor that selectively targets cancer stem cells by blocking lysosomal iron translocation, leading to lysosomal iron accumulation, ROS production, and ferroptosis (68). However, it must be noted that ferroptosis inducers have potentially harmful side effects, mainly manifested as DNA damage and cell death in normal bone marrow cells and various tissues (235, 236).

In addition to the above two strategies, another strategy is to directly target proteins related to iron homeostasis disorders. Such as methods that target TFR1, which is commonly overexpressed in tumor cell species. W. Liu et al. developed endogenous human ferritin heavy-chain nanocages (HFn) to serve as the carrier of paclitaxel (PTX), which can specifically bind to blood-brain barrier and TFR1 widely overexpressed in glioma cells, and showed that HFn-PTX showed the best anti-tumor effect, and the median survival time was significantly longer than that of free PTX (207); additionally, doxorubicin-loaded ferritin heavy chain (DOX-FTH) can be taken up and induce apoptosis of cancer cells overexpressing TFR1 (208); and Cheng et al. indicated that HFn-Dox treatment could significantly improve the therapeutic effect of doxorubicin on gastric cancer, and increase overall survival rate of tumor-bearing mice (179).

Another possible new treatment strategy is to target miRNAs that regulate iron metabolism or to use miRNA in combination with chemotherapeutic drugs. Such as, our laboratory observed that miR-375 can trigger the ferroptosis through targeting *SLC7A11*, which is essential for miR-375-mediated inhibition on gastric cancer cell stemness (209); Overexpression of miR-148a can inhibit the proliferation of liver cancer cells by targeting *TFR1* (32); MiR-184 can inhibit the occurrence and development of liver cancer by regulating the expression of *HAMP* (210); And overexpression of miR-200b can reduce ferritin levels *in vitro* and increase the sensitivity of cancer cells to doxorubicin (211).

## Perspectives and Future Directions

Iron is necessary for normal cell metabolism, while as a redox active metal, iron can also produce active oxygen, which is a potentially toxic substance. In recent years, there has been an exPLoSive growth in the research of iron homeostasis in normal cells and iron metabolism disorders in cancer. In most tumors, cancer cells retain iron metabolism pathways similar to those of normal cells, but the levels of many proteins and enzyme activities are changed, which indicates that reprogramming of iron metabolism is an important aspect of tumor cell survival. The increase of iron levels in cancer cells promotes the activity of iron-dependent proteins, while avoids the damage caused by iron overload and achieves the “adjusted iron homeostasis” in line with tumor metabolism. However, the specific mechanism is not yet fully understood, such as is the iron metabolism of different types of tumor cells consistent? How to quantify the level of iron so as to specifically target tumor cells without harming normal cells?

It may be helpful to solve these problems by developing powerful quantitative methods to measure the metabolizable iron in the cytoplasm and organelles, as well as developing iron chelating agents for organelles. Additionally, currently, the main treatment is to trigger the apoptotic death of tumor cells with anticancer drugs. However, due to the intrinsic and acquired resistance of tumor cells to apoptosis, the therapeutic effect is limited, and drug resistance is still the main limiting factor for the cure of tumor patients. Therefore, using other forms of non-apoptotic cell death provides a new therapeutic strategy. Importantly, abnormal lipid metabolism, ROS accumulation, and iron addiction are the physiological differences between malignant tumor cells and normal cells, and these differences happen to be the key regulatory factors of ferroptosis. Therefore, compared with normal cells, tumor cells are more sensitive to ferroptosis. From our own perspectives, ferroptosis inducers could have a worthwhile therapeutic index. However, based on different metabolic states and expression levels of key regulatory proteins, different tumor cells might have different sensitivity and response to ferroptosis. Notably, increasing evidences showed that combined use of ferroptosis inducer and other treatments, such as chemotherapy, radiotherapy, and immunotherapy, is a good treatment strategy, especially for cancer stem cells and drug-resistant cells. Thus, it is expected that cancer cell iron addiction will be successfully used as an effective cancer treatment in the next few years, such as: introducing drugs that induce ferroptosis into the clinic, transforming the unique iron metabolism characteristics of cancer stem cells into therapeutic advantages, and developing more targeted and effective anti-cancer iron chelating agents.

## Author Contributions

QG, SH, and ZY reviewed the literature and drafted the article. LL, XL, CL, WZ, and LZ finalized the paper and provided suggestions to improve it. All authors participated in designing the concept of this manuscript. All authors contributed to the article and approved the submitted version.

## Funding

This work was supported by the National Natural Science Foundation of China (no. 81903857), the Science and Technology Research Project of Henan Province (no. 202102310158); the Medical Science and Technology Research Project of Henan Province (no. LHGJ20190675); the Medical Science and Technology Research Project of Henan Province (no. SBGJ202003010); the 2019 Ningbo Natural Science Foundation (2019A610311); the Doctoral Research Start-up Foundation of Henan Cancer Hospital, and the Priority Academic Program Development (PAPD) of Jiangsu Higher Education Institutions.

## Conflict of Interest

The authors declare that the research was conducted in the absence of any commercial or financial relationships that could be construed as a potential conflict of interest.

## Publisher’s Note

All claims expressed in this article are solely those of the authors and do not necessarily represent those of their affiliated organizations, or those of the publisher, the editors and the reviewers. Any product that may be evaluated in this article, or claim that may be made by its manufacturer, is not guaranteed or endorsed by the publisher.

## Abbreviations

DCYTB, duodenal cytochrome B; DMT1, divalent metal transporter 1; HO-1, heme oxygenase-1; FPN1, ferroportin; HEPH, hephaestin; TF, transferrin; TFR1, transferrin receptor; STEAP3, six-transmembrane prostate epithelial antigen 3; LIP, labile iron pool; IRPs, iron-responsive element binding proteins; IRE, iron responsive element; mRNA, messenger RNA; BMP6, bone morphogenetic protein; ROS, reactive oxygen species; DFO, Desferoxamine; IREB2, Iron responsive element binding protein 2 gene; ISC, iron-sulfur cluster; GSSG, glutathione disulphide; GSH, glutathione; L-OHs, lipid alcohols; L-OOHs, lipid hydroperoxides; PUFA, polyunsaturated fatty acids; GPX4, glutathione peroxidase 4; NCOA4, nuclear receptor coactivator 4; FTH1, ferritin heavy chain 1; FTL, ferritin light chain.
